# Longitudinal Monocyte Subset Dynamics as Biomarker in Adult Histiocytosis: Association With Mutational Status, Kinase Inhibitor Exposure and Relapse Risk

**DOI:** 10.1002/hon.70233

**Published:** 2026-08-01

**Authors:** Jerome Razanamahery, Caroline Raymond, Matthias Papo, Francesco Pegoraro, Julien Guy, Ludwig Serge Aho, Jean‐François Emile, Maxime Samson, Sylvain Audia, Julien Haroche, Bernard Bonnotte

**Affiliations:** ^1^ Internal Medicine and Clinical Immunology Department Université Bourgogne Europe CHU Dijon Bourgogne INSERM U1098 Dijon France; ^2^ Internal Medicine Department Sorbonne Université Assistance Publique Hôpitaux de Paris Pitié‐Salpêtrière Hospital National Reference Center for Histiocytosis Boulogne France; ^3^ Department of Experimental and Clinical Medicine University of Florence Florence Italy; ^4^ Hematology Laboratory Université Bourgogne Europe CHU Dijon Bourgogne Dijon France; ^5^ Department of Epidemiology and Biostatistics Université Bourgogne Europe CHU Dijon Bourgogne Dijon France; ^6^ Paris‐Saclay University Versailles SQY Assistance‐Publique Hopitaux de Paris Ambroise‐Paré Hospital Smart Imaging Service de Pathology Boulogne France

**Keywords:** *BRAF* ^*V600E*^ mutation, histiocytoses, monocyte subset, relapse‐free survival, targeted therapies

## Conflicts of Interest

The authors declare no conflicts of interest.

Histiocytoses are clonal disorders driven by pathological activation of the mitogen‐activated protein kinase (MAPK) pathway (including *BRAF, MAP2K1, KRAS, NRAS*) within CD68+ histiocytes [1]. These conditions involve a complex interplay of clonal signals, immune responses, and inflammatory microenvironments. Clinical and radiological presentation are highly heterogenous depending on the histiocytosis type and mutational status. Despite significant therapeutic advances, relapses remain a challenge, particularly during treatment tapering or discontinuation [2, 3]. Recent studies underscore the critical role of monocytes in histiocytosis [4, 5].

Monocytes originate from hematopoietic stem cell progenitors and serve as essential precursors that infiltrate peripheral tissues to differentiate into macrophages (histiocytes) and dendritic cells [6]. Human monocytes are characterized by three functionally distinct subsets (i.e., classical, intermediate and non‐classical), each maintaining specific roles in both steady‐state homeostasis and diseases [7, 8]. Classical monocytes represent the primary myeloid‐derived population and are implicated in the systemic inflammatory response and hematological cancer [9]; notably, this subset is identified as the cellular vehicle for the *BRAF* ^*V600E*^ mutation in “L‐group” histiocytoses [4, 5]. Conversely, non‐classical monocytes are specialized for endothelial patrolling and vascular repair [10], while the intermediate subset is characterized by a high capacity for antigen presentation alongside pro‐inflammatory signaling [11].

Research has revealed treatment‐induced shifts in monocyte subset distribution [12] and linked intermediate monocyte levels to disease control at single time points [13]. For instance, *Papo and al* [12] reported a decrease in classical monocytes in patients treated for Erdheim‐Chester Disease (ECD); while our recent work identified a decrease in intermediate monocytes in patients with controlled disease [13]. However, comprehensive longitudinal analyses of monocyte subsets and their direct relationship to disease activity and relapse have not been explored in adult cohorts. To address this knowledge gap and to generate preliminary data on potential blood‐based biomarkers, we conducted a pilot longitudinal analysis of monocyte subset dynamics, aiming to identify factors associated with their distribution and explore links to relapse status.

**FIGURE 1:**
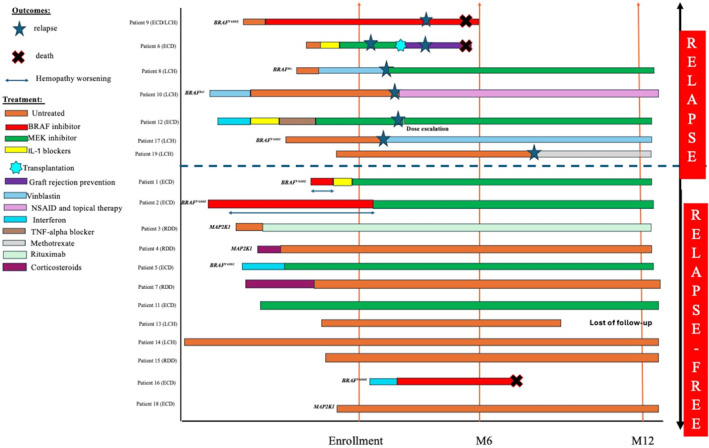
Swimmer plot of the adult patients with histiocytosis.

Our pilot study included 19 adult patients with histiocytosis: 8 Erdheim‐Chester Disease (ECD) (4 with *BRAF* ^*V600E*^ mutation), 6 Langerhans cell histiocytosis (LCH) (one with *BRAF* ^*V600E*^ mutation, 2 with exon 12 deletion), one mixed LCH/ECD (*BRAF* ^*V600E*^ mutation), and 4 Rosai‐Dorfman Disease (RDD) (2 with *MAP2K1* mutation). Diagnosis of histiocytosis, molecular testing and disease activity staging with positron emission tomography (PET‐CT) were established according to guidelines [14, 15, 16]. Histiocytosis type and disease history are available in Figure 1 and Table S1 and S2. Peripheral blood samples were collected at three standardized time points: enrollment, 6 months (M6), and 12 months (M12). Peripheral blood monocyte subsets were quantified using a standardized flow cytometry protocol adapted from the multicenter assay described by Tarfi and al [17]. Whole blood (100 μL diluted to adjust white blood cells to 15 × 10^9^/L if needed) was collected in EDTA and stained with the following antibodies: CD14‐Phycoerythrin (clone RMO52); CD16‐Brilliant Violet/BV421 (clone 3G8); CD7‐Allophycocyanin Alexa Fluor/AA700 (clone 8H8.1); CD11b‐Allophycocyanin Alexa Fluor/AA750 (clone Bear1); CD13‐Phycoerythrin DyLight 594 (clone WM15); CD15‐Fluorescein isothiocyanate/FITC (clone 80H5); CD33‐Phycoerythrin Cyanin 5.5/PC5.5 (clone D3HL60.251); CD45‐Krome Orange/KO (clone J33). Samples were acquired following a lyse/no wash procedure (IOT 1X, Beckman Coulter) on a Navios cytometer (Beckman‐Coulter) and analyzed with Kaluza software (Beckman‐Coulter). Monocytes were roughly selected as CD45^high^/SSC (side scatter)^int^. Monocytes were defined as CD45^+^/SSC and CD33^+^/SSC intermediate cells. To ensure the exclusion of contaminating populations, **Lineage‐Negative Exclusion sequential** gates were applied. Immature and mature granulocytes were identified as CD45^int^/CD16^high^ cells or SSCint‐to‐high cells and T lymphocytes expressing CD7 were excluded. The remaining CD14^−^CD16^−^ cells correspond mainly to basophils and residual lymphocytes. The resulting pure monocyte population was projected onto CD14/CD16 scattergram and divided into three functional subsets according to the international nomenclature: classical (**cMo**:CD14^++^/CD16^−^ intermediate (**iMo:** CD14^++^/CD16^+^), and non‐classical (**ncMO**: CD14^+^/CD16^++^ following established nomenclature [18]. A minimum of 10.000 monocytes events were required for analysis to ensure the precision of the classical monocyte fraction. The full gating procedure is detailed in Figure S1.

Quantitative variables were compared using the Mann‐Whitney test (reported as median with [IQR]) and categorical variables using Chi‐square or Fisher's exact tests (reported as *n* [%]), as appropriate. Longitudinal changes were assessed via mixed‐model ANOVA for repeated measures with Tukey's post‐hoc test. Relapse predictors were evaluated using time‐adjusted univariate logistic regression; variables with *p* < 0.2 were entered into a multivariable model. LASSO regression was applied for variable selection to prevent overfitting. Findings were validated using a Bayesian random‐effects logistic regression model (Gibbs sampling: 2500 burn‐in, 10,000 MCMC iterations), with convergence assessed by Gelman‐Rubin diagnostics. Analyses were performed using GraphPad Prism V.10 and Stata Now 18.5, with significance defined as *p* < 0.05. The study received local ethics committee approval and adhered to the Declaration of Helsinki principles.

**FIGURE 2:**
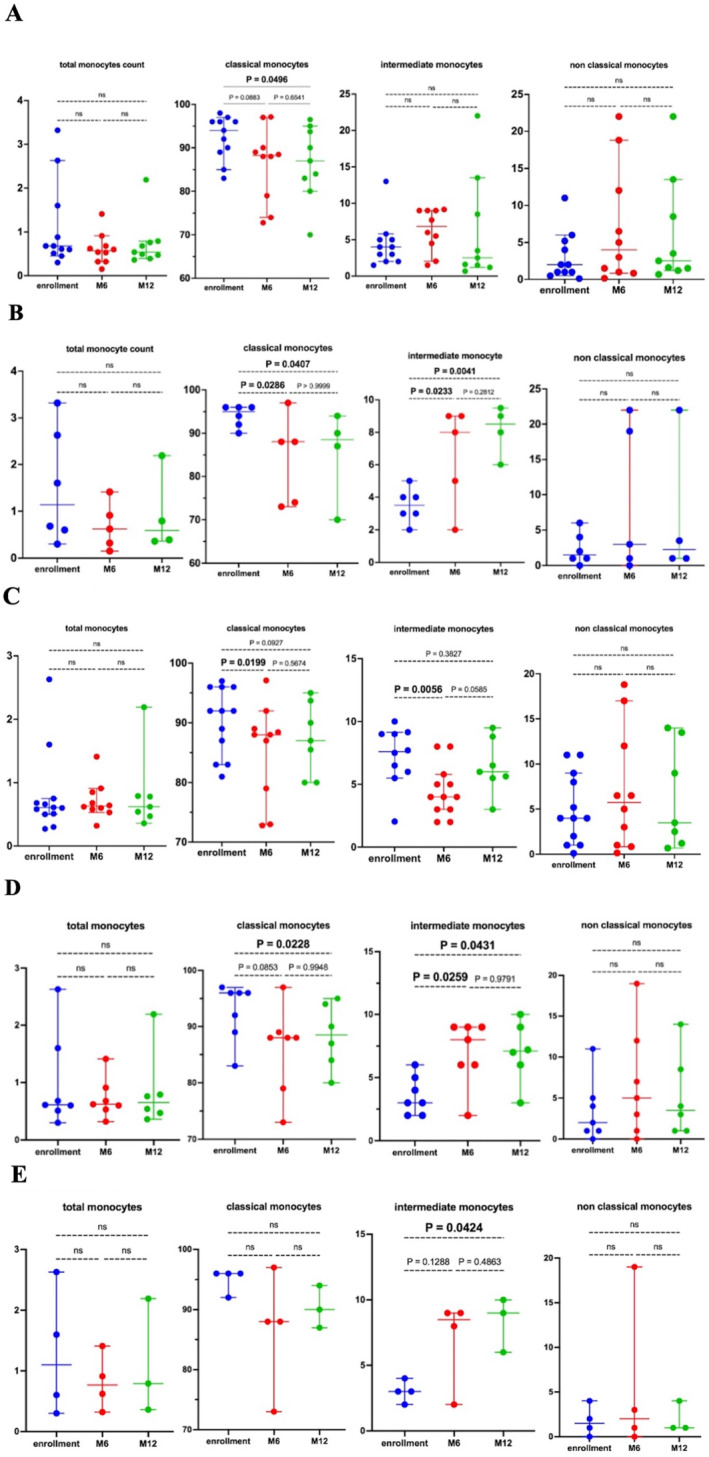
Monocyte subset dynamic over time in adults histiocytosis: association with mutational status and relapse. (A) Longitudinal analysis of monocyte subsets in MAP‐kinase mutated patients (*n* = 11). (B) Longitudinal analysis of monocyte subsets in BRAFV600 E mutated patients (*n* = 6). (C) Longitudinal analysis of monocyte subsets in all non‐relapsing patients (*n* = 12). (D) Longitudinal analysis of monocyte subsets in non‐relapsing patients with MAP‐kinase mutations (*n* = 7). (E) Longitudinal analysis of monocyte subsets in non‐relapsing patients with *BRAF* ^*V600E*^ mutations (*n* = 4) treated with targeted therapies.

In MAP‐kinase mutated patients (*n* = 11), classical monocytes decreased from enrollment to M12, with a mean decrease of 5.66% points (95% CI [0.01 to 11.28]; *p* = 0.049) (Figure 2A). This decline was primarily observed in *BRAF* ^*V600E*^ patients (*n* = 6), who showed a mean decrease of 10% points in classical monocytes from enrollment to M6 (95% CI [1.236 to 18.76]; *p* = 0.028) and 10.01% points to M12 (95% CI [0.49 to 19.52]; *p* = 0.04) Concurrently, these *BRAF* ^*V600E*^ patients exhibited a mean increase in intermediate monocytes of 3.23% points from enrollment to M6 (95% CI [−5.94 to −0.526]; *p* = 0.023) and 4.93% points to M12 (95% CI [−7.86 to −1.99]; *p* = 0.004) (Figure 2B). This pattern, particularly the decrease in classical monocytes, may reflect a reduction in mutational burden, aligning with disease control, as *BRAF* ^*V600E*^ mutations have been previously identified in CD14+ classical monocytes in histiocytosis [5].

We did not observe any longitudinal differences in monocyte subsets according to histiocytosis type, clinical phenotype (cardiovascular or neurological involvement), disease extent (unicentric vs. multicentric), baseline C‐reactive protein elevation, or the presence of a bone marrow mutation.

During follow‐up, 7 patients experienced relapse (4 LCH, 2 wild‐type ECD, 1 *BRAF* ^*V600E*^ mixed ECD/LCH). The relapse‐free group (*n* = 12) included 6 ECD, 4 RDD, and 2 isolated pulmonary LCH patients. Patient characteristics, including histiocytosis type, disease extent, mutational status, and pre‐enrollment treatment, were comparable across groups. Notably, relapse‐free patients had a significant mean decrease of 5.66% points in classical monocytes (95% CI [0.36 to 10.96]; *p* = 0.02) and 3.06% points in intermediate monocytes (95% CI [1.29 to 4.82]; *p* = 0.006) between enrollment and M6 (Figure 2C). This reduction in intermediate monocytes was more pronounced in relapse‐free patients during this period (median [IQR]: 4 [−4 to −2] vs. 2 [−3 to 8]; *p* = 0.017). No significant changes were observed in relapsing patients. All other cell lineages and CRP levels remained comparable across groups.

Within the relapse‐free group, MAP‐kinase mutated patients (*n* = 7) also demonstrated a decrease in classical monocytes from enrollment to M12 (mean decrease of 4.5% points; 95% CI [−8.11to −0.96]; *p* = 0.0228) and an increase in intermediate monocytes from enrollment to M6 (mean increase of 3.42% points; 95% CI [0.52 to 6.33]; *p* = 0.02) and M12 (mean increase of 3.53% points; 95% CI [0.47 to 10.4]; *p* = 0.04) (Figure 2D). Similarly, *BRAF* ^*V600E*^ patients (*n* = 4), all receiving targeted therapies, exhibited an increased intermediate monocyte population from enrollment to M12 (mean increase of 5.66% points; 95% CI [0.47 to 10.8]; *p* = 0.04) (Figure 2E).

Time‐adjusted logistic regression analysis, identified an association between relapse risk and intermediate monocyte percentage (OR: 0.51; 95% CI [0.27 to 0.96]; *p* = 0.038). Bayesian logistic regression confirmed these findings, identifying intermediate monocytes (Median effect on relapse: −41; 95% CI [−173 to 202]) as the main biological predictor of relapse.

This pilot study offers initial insights into the longitudinal dynamics of monocyte subsets in adult histiocytosis, linking their patterns to MAPK mutational status, targeted therapy, and relapse risk. In MAP‐kinase‐mutated patients, particularly those with *BRAF* ^*V600E*^ mutations, targeted therapy was associated with a decrease in classical monocytes and a concomitant increase in intermediate monocytes. This decrease in classical monocytes may reflect a reduction in mutational burden, aligning with disease control, as *BRAF* ^*V600E*^ mutation has been previously identified in CD14+ classical monocytes [5].

Crucially, our findings indicate that relapse‐free patients experienced an early decrease in both classical and intermediate monocytes, potentially signaling a shift toward a less inflammatory immune profile. The dual role of intermediate monocytes (CD14++CD16+), which can exert both pro‐inflammatory and anti‐inflammatory effects [7], suggests that their dynamics could be a critical indicator of disease activity. The subgroup of patients treated with targeted therapy (mostly MEK‐inhibitor) exhibited a distinct recovery of the intermediate subset. This may

Represent a context‐dependent immune adaptation, influenced by the specific disease type (notably ECD) and mutation profile, consistent with recent report of lineage shift of *BRAF* ^*V600E*^ mutant myeloid cells under MEK‐inhibition [19].

The identification of intermediate monocyte fraction as a predictor of relapse risk (OR: 0.51; *p* = 0.038) is a significant finding, and its confirmation with a Bayesian analysis strengthens its potential value as simple circulating biomarker for follow‐up. This circulating biomarker appears independent of CRP levels, which is clinically relevant because classical inflammatory markers can be absent in many histiocytosis patients. Monocyte immunophenotyping may therefore capture a distinct pathophysiological signal related to from myeloid lineage shift rather than merely reflecting systemic inflammation. Consequently, intermediate monocyte monitoring could offer clinical value even in patients with normal CRP. This finding seems of particular interest because, unlike in some pediatric cohorts [20], mutational status did not solely influence relapse risk.

It is imperative to underscore the pilot nature and limitations of this study, including its small, heterogeneous cohort and single‐center design. The variability in histiocytosis subtypes, molecular backgrounds, and prior treatments necessitates cautious interpretation. While these preliminary observations generate important signals, their generalizability is limited. Future, larger multicenter studies focusing on more homogeneous patient groups are essential to validate these findings rigorously, define specific thresholds for monocyte subsets, and clarify their precise clinical utility. Further single‐cell functional analyses are also needed to fully delineate the roles of each monocyte subset in histiocytosis.

Despite these limitations, this proof‐of‐concept study reinforces the importance of the monocyte/macrophage axis in histiocytic disorders.

Our data suggest that longitudinal monitoring of monocyte subsets may complement to current imaging‐based assessments and offer new avenues for personalized disease management and follow‐up in clinical practice, particularly in scenarios of treatment de‐escalation or discontinuation.

## Author Contributions

J.R., J.H., B.B.: study design. J.G., J.R.: flow cytometry analysis. J.‐F.E.: pathology review and molecular status determination for histiocytoses. J.R., L.S.A.: statistical analysis. J.H., M.P., F.P., C.R., M.S., S.A.: clinical expertise. All authors edited the manuscript and approved the final version of the manuscript for submission.

## Funding

The authors have nothing to report.

## Supporting information


Supporting Information S1

## Data Availability

Complete data including flow cytometry analysis can be requested from the corresponding author at: razanamahery.jerome@hotmail.fr.
