# Supplementary material for: Longitudinal Monocyte Subset Dynamics as Biomarker in Adult Histiocytosis: Association With Mutational Status, Kinase Inhibitor Exposure and Relapse Risk
Source: Hematol Oncol. 2026 Aug 1;44(5):e70233. doi: 10.1002/hon.70233 (PMC13428496; doi:10.1002/hon.70233)
Supplement: Supplementary file 1 — Supporting Information S1 [file HON-44-e70233-s001.docx]

**Flow cytometry analysis**

Peripheral blood monocyte subsets were quantified using a standardized flow cytometry protocol adapted from the validated multicenter assay described by **Tarfi et al. (2018).** Whole blood (100 μL diluted to adjust white blood cells to 15 × 10^9^/L if needed) were collected on EDTA and stained with the following antibodies: CD14-Phycoerythrin (clone RMO52); CD16-Brilliant Violet/BV421 (clone 3G8); CD7-Allophycocyanin Alexa Fluor/AA700 (clone 8H8.1); CD11b-Allophycocyanin Alexa Fluor/AA750 (clone Bear1); CD13-Phycoerythrin DyLight 594 (clone WM15); CD15-Fluorescein isothiocyanate/FITC (clone 80H5); CD33-Phycoerythrin Cyanin 5.5/PC5.5 (clone D3HL60.251); CD45-Krome Orange/KO (clone J33). Samples were acquired following a lyse/no wash procedure (IOT 1X, Beckman Coulter) procedure on a Navios cytometer (Beckman-Coulter) and analyzed with Kaluza software (Beckman-Coulter). Monocytes were roughly selected as CD45^high^/SSC(side scatter)^int^. Monocytes were defined as CD45^+^/SSC and CD33^+^/SSC intermediate cells. To ensure the exclusion of contaminating populations, **Lineage-Negative Exclusion** sequential gates were applied. The immature and mature granulocytes as CD45^int^/CD16^high^ or **SSCint-to-high** cells and T lymphocytes expressing CD7 were then excluded. The remaining CD14^−^CD16^−^ cells correspond mainly to basophils and residual lymphocytes. The resulting pure monocyte population was projected onto a **CD14/CD16 scattergram** and subdivided into three functional subsets according to international nomenclature: **classical (cMo: CD14++/CD16−)**, **intermediate (iMo: CD14++/CD16+)**, and **non-classical (ncMo: CD14+/CD16++)** as previously described^1^ (**Figure 1**). A minimum of 10,000 monocyte events was required for analysis to ensure the precision of the classical monocyte fraction.

The samples were obtained at a steady state for histiocytosis, after ruling out infection and hemopathy worsening.

**Diagnosis procedure for histiocytosis**

All histiocytosis were diagnosed based on the current guidelines and the presence of histiocytic infiltration within tissue biopsy^2,3^. All tissue biopsies were centralized and reviewed by Professor Jean-François Emile (Ambroise-Paré Hospital).

Histology was performed on 4-micrometer thick tissue sections after staining with Hematoxylin & Eosin and immunohistochemistry, including at least CD1a, S100, and CD68, CD163 primary antibodies. Detection of mutations was performed on tissue biopsies infiltrated by histiocytosis. Tumor DNA was extracted from formalin-fixed and paraffin-embedded tissues. Detection of *BRAF^V600E^* mutation was performed as previously described using picodroplet digital PCR^4,5^. Detection of other mutations was performed using targeted next-generation sequencing (NGS). Samples were analyzed using MiSeq (Illumina^®^) after preparing the Custom Amplicon Low Input Kit libraries. The targeting genes are listed as follows: *AKT1, ALK, ARAF, ASXL1, BRAF, CALR, CBL, CDK4, CDKN1B, CDKN2A, CEBPA, CSF3R, CTNNB1, DNMT3A, EGFR, EZH2, FLT3, GATA2, GNA11, GNAQ, GNAS, HERC1, HRAS, IDH1, IDH2, JAK2, JAK3, KIT, KRAS, KTM2D, MAML3, MAMLD1, MAP2K1, MAP2K2, MAP2K3, MAP2K4, MAP2K6, MAP3K1, MAP3K8, MAP3K9, MAP3K10, MAP3K19, MAP4K4, MAPK1, MAPK11, MAPK9, MPL, NF1, NOTCH1, NOTCH2, NPM1, NRAS, PDGFRA, PIK3CA, PP6C, PTEN, PTPN11, RAC1, RAF1, RIT1, RUNX1, SETBP1, SRSF2, STAG2, STK19, SYNGAP1, TAOK1, TAOK2, TET2, TP53, U2AF1, WT1, ZRSR2.*

Erdheim-Chester disease (ECD) patients had iconic lesions including long bone involvement and/or perinephric fat infiltration and/or vascular sheathing of the adventitia of vessels with compatible histology showing tissue infiltrate by CD68^+^, CD1a^-^ histiocytes^6,7^. Tissue biopsy presented gain of function mutation in genes of the MAP-kinase pathway (if available) or at least moderate to strong expression of phosphorylated-Erk^6,7^.

Rosai-Dorfman Disease (RDD) patients had typical histology with tissue biopsy disclosing infiltration by CD68^+^, S100^+^, and CD1a^-^ histiocytes with enlarged nuclei and lesions of emperipolesis^8^.

Langerhans Cell Histiocytosis (LCH) patients had a biopsy showing an inflammatory infiltrate associated with CD68^+^, CD1a+, S100^+^ histiocytes or typical pulmonary images (after excluding differential diagnosis) and compatible broncho-alveolar lavage in the absence of tissue biopsy^9^.

All patients had at least a ^18^fluorodeoxyglucose positron emission tomography at diagnosis to establish specific organ involvement.

The disease activity at each visit was assessed through a physical examination, complete biological test and a ^18^ FDG PET/CT as recommended by experts recommendations. Relapse was defined by worsening of a skin disease or a progressive metabolic response on imaging according to PERCSIT criteria^10^.

**Detection of myeloid neoplasm driver gene:**

The myeloid gene panel analyzed by next generation sequencing (NGS) included: *ASXL1, BCOR, BCORL1, CALR, CBL, CSF3R, DNMT3A, ETV6, EZH2, FLT3, GATA2, IDH1, IDH2, JAK2, KIT, KRAS, MPL, NIPBL1, NPM1, NRAS, PHF6, PTPN11, RAD21, RIT1, RUNX1, SETBP1, SF3B1, SMC1A, SMC3, SRSF2, STAG2, TET2, TP53, U2AF1, WT1, ZRSR2*. Variant allele frequency (VAF) was considered significant over a threshold of 2%^11^. The assessment was performed on blood samples or bone marrow aspiration.

**Statistical analysis**

Quantitative data were expressed by a median with an interquartile range and were compared between groups with the Mann-Whitney test. Qualitative data are expressed with numbers (percentage) and compared using Chi2 or Fischer exact test as appropriate. For repetitive data analysis, we have used an analysis of variance test (ANOVA test) with a mixed model of repeated measure followed by a Tukey’s multiple comparison test. All reported p-values were two-sided, and a p-value <0.05 was considered statistically significant. The logistic regression model aimed to evaluate variable associated with relapse. The independent variable included in the regression model were: hemoglobin, white count cells, neutrophils, lymphocytes, C-reactive protein, monocytes counts, classical, intermediate, and non-classical monocytes, ECD, RDD, LCH, unicentric disease, widespread disease, BRAF mutation, myeloid neoplasm driver gene mutation. All biological variables were reported at enrollment, month 6 and month 12. A univariate logistic regression model was performed to evaluate the association with relapse with an adjustment with time for repetitive variable. The multiple regression model was performed with all variables with a p-value<0.2 in univariate regression analysis. Lasso regression was performed to prevent overfitting (variable selection). A Bayesian random effects logistic regression model was constructed to investigate the impact of variables on relapse risk. The model included all aforenoted variable as covariates. Gibbs sampling was used for variance components with a burn-in of 2,500 iterations and 10,000 MCM sample size. Model convergence was assessed using trace plots and the Gelman-Rubin diagnostic. For all graph analyses, significant p-values are presented as follows: *p<0.05; **p<0.001. Statistical analysis was performed using GraphPad software, V.10 (GraphPad, San Diego, California, USA) and StataNow 18.5 software.

**Reference:**

1. Ziegler-Heitbrock L, Ancuta P, Crowe S, et al. Nomenclature of monocytes and dendritic cells in blood. *Blood*. 2010;116(16):e74-80.

2. Emile J-F, Cohen-Aubart F, Collin M, et al. Histiocytosis. *Lancet*. 2021;

3. Emile J-F, Abla O, Fraitag S, et al. Revised classification of histiocytoses and neoplasms of the macrophage-dendritic cell lineages. *Blood*. 2016;127(22):2672–2681.

4. Diamond EL, Durham BH, Haroche J, et al. Diverse and Targetable Kinase Alterations Drive Histiocytic Neoplasms. *Cancer Discov*. 2016;6(2):154–165.

5. Melloul S, Hélias-Rodzewicz Z, Cohen-Aubart F, et al. Highly sensitive methods are required to detect mutations in histiocytoses. *Haematologica*. 2019;104(3):e97–e99.

6. Goyal G, Heaney ML, Collin M, et al. Erdheim-Chester disease: Consensus recommendations for the evaluation, diagnosis, and treatment in the molecular era. *Blood*. 2020;

7. Haroche J, Cohen Aubart F, Amoura Z. Erdheim-Chester disease. *Blood*. 2020;

8. Abla O, Jacobsen E, Picarsic J, et al. Consensus recommendations for the diagnosis and clinical management of Rosai-Dorfman-Destombes disease. *Blood*. 2018;131(26):2877–2890.

9. Goyal G, Tazi A, Go RS, et al. Expert consensus recommendations for the diagnosis and treatment of Langerhans cell histiocytosis in adults. *Blood*. 2022;blood.2021014343.

10. Wahl RL, Jacene H, Kasamon Y, Lodge MA. From RECIST to PERCIST: Evolving Considerations for PET response criteria in solid tumors. *J Nucl Med*. 2009;50 Suppl 1(Suppl 1):122S–50S.

11. Steensma DP, Bejar R, Jaiswal S, et al. Clonal hematopoiesis of indeterminate potential and its distinction from myelodysplastic syndromes. *Blood*. 2015;126(1):9–16.

**Figure S 1**: Gating strategy for the identification of monocyte subsets.


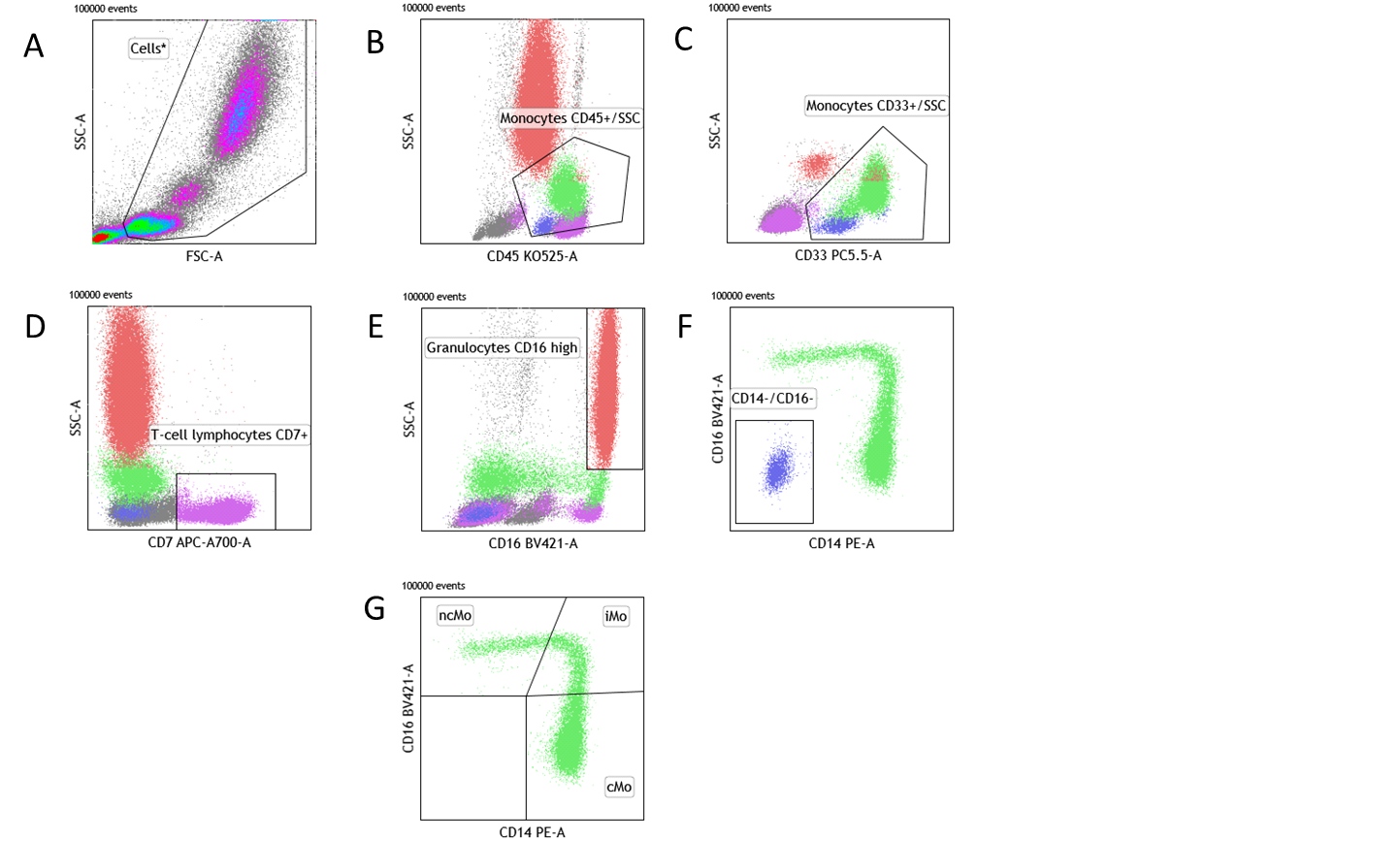


Detailed hierarchical gating strategy used to identify peripheral blood monocyte subsets, adapted from the multicenter-validated "monocyte assay".

**(A) Morphological selection :** Initial identification of the cell population was performed using Forward Scatter (FSC) and Side Scatter (SSC) parameters.

**(B) Primary Monocyte Gate:** A "rough" monocyte population was first identified based on **CD45 high** expression and intermediate Side Scatter (**SSC int**).

**(C) Myeloid Marker Validation:** To refine the selection, the population was further gated for the expression of the myeloid marker **CD33**, distinguishing monocytes from other lymphoid contaminants.

**(D-F) Lineage-Negative Exclusion Strategy (Cleanup Gates):** To ensure a pure monocyte population before subset subdivision, sequential exclusion gates were applied:

- **(D) T-lymphocyte exclusion:** CD7+ cells were gated out to remove T-cell contamination.
- **(E) Granulocyte exclusion:** Residual mature and immature granulocytes were excluded based on high CD16 expression (**CD16 high**) and high SSC.
- **(F) Non-monocytic cell exclusion:** Remaining non-monocytic cells, including basophils and residual lymphocytes, were identified as **CD14−/CD16−** cells and excluded.

**(G) Total Monocyte Population:** The resulting "lineage-negative" population (CD45+/CD33+/CD7−/CD16−/int) represents the purified total monocyte population.

**Subsets Subdivision:** Finally, monocytes were projected onto a **CD14/CD16 scattergram** and subdivided into three functional subsets according to international nomenclature: **Classical monocytes (cMo):** CD14++/CD16−, **Intermediate monocytes (iMo):** CD14++/CD16, **Non-classical monocytes (ncMo):** CD14+/CD16++.

.

**Table S 1:** Characteristics of the patients

| **Case** | **age at dosage** | **type of histiocytosis** | **location of histiocytosis** | **mutation BRAF** | **mutation MAP2K1** | **Hematological malignancy** | **Clonal hematopoiesis** | **mutation in bone marrow with NGS** |
| --- | --- | --- | --- | --- | --- | --- | --- | --- |
| #1 | 71 | ECD | mesentery, bone, CNS, peri-renal, heart | *c,1799T>A,p,(Val600Glu),* | *0* | CMML | 0 | *TET2/ZRS2/KRAS/NRAS/BRAF* |
| #2 | 72 | ECD | mesentery, bone, CNS, peri-renal, heart, bone marrow | *c,1799T>A,p,(Val600Glu),* | *0* | CMML | 0 | *TET2/SRSF2/CLB/NRAS* |
| #3 | 71 | RDD | perirenal | *0* | *c,395C>T, p(Ala132Val)* | 0 | 0 | *0* |
| #4 | 41 | RDD | skin,bone,eyes, vessels | *0* | *c,361T>A,p(Cys121Ser)* | 0 | 0 | *0* |
| #5 | 73 | ECD | heart, bones, vessels, mesentery, peri-renal | *c,1799T>A,p,(Val600Glu),* | *0* | ET | 0 | *JAK2/TET2/NF1* |
| #6 | 25 | ECD | vessels, bone, sinus | *0* | *0* | 0 | 0 | *0* |
| #7 | 67 | RDD | bone | *0* | *0* | 0 | 0 | *0* |
| #8 | 64 | LCH | liver, endocrine, bone, skin | *c,1457_1471del,p,(486_490del)* | *0* | 0 | 1 | *TET2* |
| #9 | 77 | LCH/ECD | bone, lung , skin, vessels | *c,1799T>A,p,(Val600Glu),* | *0* | 0 | 1 | *KRAS/SH2B3/SRSF2/TET2* |
| #10 | 22 | LCH | bone, skin, endocrine, lung | *c,1457_1471del,p,(486_490del)l* | *0* | 0 | 0 | *0* |
| #11 | 66 | ECD | bone, peri-renal, mesentery | *0* | *0* | 0 | 1 | *TET2* |
| #12 | 63 | ECD | bone, mesentery, peri-renal | *0* | *0* | 0 | 1 | *DNMT3A* |
| #13 | 68 | LCH | lung | *0* | *0* | *0* | 0 | *0* |
| #14 | 44 | LCH | lung, hypophysis, bones | *0* | *0* | 0 | 0 | *0* |
| #15 | 65 | RDD | skin, lymph node | *0* | *0* | 0 | 0 | *0* |
| #16 | 81 | ECD | bone, heart, vessel, CNS, peri-renal | *c,1799T>A,p,(Val600Glu),* | *0* | 0 | 1 | *ASXL1/NF1/TET2/U2AF1* |
| #17 | 63 | LCH | bone, lung, pituitary gland | *c,1799T>A,p,(Val600Glu),* | *0* | 0 | 0 | *0* |
| #18 | 74 | ECD | mesentery, peri-renal | *0* | *c,209A>G, p(Lys97Arg)* | *0* | 0 | *0* |
| #19 | 80 | LCH | skin | *0* | *0* | 0 | 1 | *ASXL1/TET2/SRSF2* |

ECD : Erdheim-Chester Disease, LCH : Langerhans Cell histiocytosis, RDD: Rosai-Dorfman Disease, CHIP: Clonal hematopoiesis of indeterminate potential, NGS: Next generation sequencing, CMML: chronic myelomonocytic leukemia, ET: essential thrombocythemia

**Table S 2:** Characteristics of the patients

| **Case** | **Prior treatment** | **Treatment at enrollment** | **monocytes counts** | **Classical monocytes** | **Intermediate monocytes** | **Non classical monocytes** | **Treatment at month 6** | **Monocytes counts** | **Classical monocytes** | **Intermediate monocytes** | **Non classical monocytes** | **Treatment at month 12** | **monocytes counts** | **Classical monocytes** | **Intermediate monocytes** | **Non classical monocytes** | **relapse** | **Targeted therapy exposure during follow-up** |
| --- | --- | --- | --- | --- | --- | --- | --- | --- | --- | --- | --- | --- | --- | --- | --- | --- | --- | --- |
| #1 | Vemurafenib | KINERET | 2,63 | 96% | 3% | 1% | Cobimetinib | 1,41 | 88% | 9% | 1% | Cobimetinib | 2,19 | 90% | 9% | 1% | 0 | 1 |
| #2 | Vemurafenib | none | 1,6 | 96% | 2% | 2% | Cobimetinib | 0,91 | 97% | 2% | 0% | Cobimetinib | 0,79 | 94% | 6% | 1% | 0 | 1 |
| #3 | Rituximab | rituximab | 0,51 | 83% | 5% | 11% | Rituximab | 0,53 | 79% | 9% | 12% | Rituximab | 0,54 | 80% | 7% | 14% | 0 | 0 |
| #4 | steroids | none | 0,61 | 97% | 2% | 1% | none | 0,6 | 88% | 6% | 7% | none | 0,47 | 95% | 3% | 2% | 0 | 0 |
| #5 | none | Cobimetinib | 0,3 | 96% | 3% | 0% | Cobimetinib | 0,32 | 88% | 9% | 3% | Cobimetinib | 0,36 | 87% | 9% | 4% | 0 | 1 |
| #6 | none | Kineret | 0,62 | 92% | 7% | 1% | Cobimetinib  then transplant reject treatment | 0,33 | 98% | 1% | 1% | NA | NA | NA | NA | NA | 1 | 1 |
| #7 | none | none | 0,66 | 92% | 4% | 4% | none | 0,64 | 92% | 8% | 1% | none | 0,62 | 86% | 6% | 9% | 0 | 0 |
| #8 | none | vinblastin | 0,88 | 85% | 13% | 2% | Cobimetinib | 0,32 | 90% | 9% | 1% | Cobimetinib | 0,68 | 83% | 16% | 2% | 1 | 1 |
| #9 | Vemurafenib | Vemurafenib | 3,32 | 94% | 5% | 1% | NA | NA | NA | NA | NA | NA | NA | NA | NA | NA | 1 | 0 |
| #10 | none | none | 0,45 | 98% | 2% | 1% | none | 0,54 | 97% | 2% | 2% | none | 0,49 | 96,50% | 1,90% | 1,60% | 1 | 0 |
| #11 | Cobimetinib | Cobimetinib | 0,27 | 81% | 8% | 11% | NA | NA | NA | NA | NA | NA | NA | NA | NA | NA | 0 | 0 |
| #12 | Interferon/Kineret/infliximab/ Cobimetinib | none | 0,68 | 71% | 25% | 4% | none | 0,68 | 84% | 8% | 9% | none | 0,65 | 92% | 5% | 3% | 1 | 0 |
| #13 | none | none | 0,58 | 92% | 4% | 4% | NA | NA | NA | NA | NA | NA | NA | NA | NA | NA | 0 | 0 |
| #14 | none | none | 0,75 | 83% | 8% | 9% | none | 0,85 | 73% | 10% | 17% | none | 0,78 | 80% | 6% | 14% | 0 | 0 |
| #15 | none | none | 0,5 | 87% | 5% | 8% | none | 0,58 | 87% | 7% | 7% | NA | NA | NA | NA | NA | 0 | 0 |
| #16 | none | none | 0,6 | 92% | 4% | 4% | Vemurafenib | 0,62 | 73% | 8% | 19% | NA | NA | NA | NA | NA | 0 | 1 |
| #17 | none | none | 0,68 | 90% | 4% | 6% | Vinblastine | 0,15 | 74% | 5% | 22% | vinblastin | 0,39 | 70% | 8% | 22% | 1 | 0 |
| #18 | none | none | 0,68 | 89% | 6% | 5% | none | 0,68 | 89% | 6% | 5% | none | 0,76 | 84% | 7,2 | 8,5 | 0 | 0 |
| #19 | none | none | 1,23 | 89% | 8% | 3% | Methotrexate | 1,41 | 86% | 8% | 3% | methotrexate | 0,57 | 80% | 12% | 8% | 1 | 0 |

#6: No M12 data available because the patient experienced a severe relapse leading to organ transplant and secondary death by fungal infection.

#9: No M6 and M12 data because the patient died from Covid-19 infection

#11: No M6 and M12 data because of technical issue during the phenotyping procedure

#13: No M6 and M12 data because of loss of follow-up

# 15: No M12 data because of loss of follow-up

#16: No M12 data because the patient died from pulmonary embolism
